# Tuning the Surface Charge of Self-Assembled Polydiacetylene Vesicles to Control Aggregation and Cell Binding

**DOI:** 10.3390/bios10100132

**Published:** 2020-09-24

**Authors:** Anthony David Nelson, Priyanka Shiveshwarkar, Butaek Lim, Gumaro Rojas, Izele Abure, Anura Shrestha, Justyn Jaworski

**Affiliations:** Department of Bioengineering, University of Texas at Arlington, 500 UTA Blvd., Arlington, TX 76013, USA; anthony.nelson2@mavs.uta.edu (A.D.N.); priyanka.shiveshwarkar@mavs.uta.edu (P.S.); butaek.lim@mavs.uta.edu (B.L.); gumaro.rojas@mavs.uta.edu (G.R.); izele.abure@mavs.uta.edu (I.A.); anura.shrestha@mavs.uta.edu (A.S.)

**Keywords:** polydiacetylene, vesicles, surface charge, pH

## Abstract

Polydiacetylene vesicles of various compositions were assembled using a two-part mixture of 10,12-pentacosadiynoic acid (PCDA) and ethylenedioxy-bis-ethylamine (EDEA)-labeled PCDA in order to control surface charge and stability within a desired pH range. Investigation of the interaction of the vesicles with mammalian cells as a function of surface charge was carried out and identified a clear correlation in cell–vesicle association and corresponding cell death for vesicles with positive surface charge. The binding behavior of the vesicles was found to be tunable by regulating the proportion of anionic PCDA relative to cationic PCDA–EDEA content within vesicles as to control the surface charge as a function of pH. Association of vesicles with cells thus depended on the corresponding charge of the vesicles and cell surface. The prospect of this work may serve as a step toward future vesicle designs to allow triggered uptake of vesicles locally within low pH tumor microenvironments.

## 1. Introduction

Several earlier works thoroughly investigated the potential of 10,12-pentacosadiynoic acid (PCDA) as an amphiphilic building block for the fabrication of self-assembled vesicles that reveal a notable visible color-shift offering it as a popular platform for sensor and assay development [1,2,3,4,5,6,7,8,9]. Vesicles constructed from PCDA exhibit a blue color after exposure to UV light and provide a negative surface charge that imparts them with colloidal stability at physiological pH. These blue vesicles can be exposed to stimuli (such as heat) that can result in a shift to a visible red color due to side-chain rearrangement causing rotation of the conjugated pi-backbone [3]. The ability of the vesicles to aggregate after exposure to a multivalent target have also been examined in detail and demonstrates another useful property of this material for agglutination based sensing applications. In this work, we constructed complex vesicles comprised of a range of mixtures of carboxylic acid terminated (PCDA) and amine terminated (PCDA–EDEA) amphiphiles in order to examine the link between the composition, colloidal stability, and surface charge response to pH.

Several previous studies have shown that polydiacetylene vesicles can exhibiting a color change in response to pH and have also revealed the ability to tune the release rate of cargo within the vesicles, specifically showing that a lower pH environment can increase the release rate [10]. The surface charge changes of polydiacetylene that follow exposure to different pH environments are said to be a major contributor to the pH responsiveness of such vesicles and depends largely upon their functional head-groups. In looking at surface charges for a two-component amphiphile system of PCDA and a phosphatidylcholine, studies have shown that the zeta potential of the polydiacetylene vesicles could be tuned from −3 to −25 mV by altering their composition [11]. Similar studies of multicomponent PCDA vesicles using mixtures of PCDA with phosphatidylcholines have shown zeta potentials from −21 to −37 mV [12]. Work from the same groups have also explored decorating the vesicles with folic acid as to provide a mechanism for interactions with folate receptors present on cells. In prior works including our own in which peptides were displayed on polydiacetylene vesicles, it has been shown that modifying the surface could provide targeted binding to cell surface integrins [13,14]. Mannose-coated polydiacetylene vesicles have likewise been developed for targeting cells, and the various studies of polydiacetylene with cultured cells have shown a notably high cell viability even at up to 1 mM [15]. The anionic charge on polydiacetylene nanoparticles made of conventional PCDA is likely a contributor to their low cytotoxicity. Cell uptake studies have shown a relatively low uptake of anionic and neutral PDA vesicles while in contrast a high uptake of cationic polydiacetylene nanoparticles has been reported [16]. The route of uptake of anionic polydiacetylene into cells has been found to occur via macropinocytosis, clathrin-dependent endocytosis, and to a great extent by caveolae-mediated endocytosis [17]. Cationic particles are also known to be taken up by more than one endocytic pathway, where incorporating pH sensitive amine motifs can allow endocytosed particles to be released as a result of their buffering capacity [18]. This effect has been widely examined across different pH sensitive moieties and charge groups for the purpose of optimizing gene transfection, and among them cationic polydiacetylene micelles have also proven to be an effective transfection reagent [19]. Such studies have suggested photopolymerization of cationic polydiacetylene vesicles to enhance transfection efficiency and provide some reduction in cytotoxicity, while others utilizing quaternary ammonium head groups have shown no beneficial effect of polymerization on the viability of HEK293 cells but did show an improvement in gene transfection for photopolymerized vesicles [20]. Mixtures of cationic polydiacetylene and phospholipids have also shown high rates of vesicle internalization in an example using the breast carcinoma cell line Bcap-37 [21]. Extending the application of polydiacetylenees to in vivo studies for tumor imaging and drug delivery applications, polyethylene glycol (specifically PEG2000) modified polydiacetylene vesicles have shown good in vivo stability and delayed phagocytosis with low toxicity even at 100 mg/kg in rodent models [22,23]. While such PEGylated polydiacetylene vesicles have been suggested to accumulate passively in tumors, more sophisticated approaches utilizing polydiacetylene nanoparticles functionalized with targeting moieties have shown more specific uptake by cells overexpressing biotin receptors [24]. These efforts by the research community show the promising avenue for polydiacetylene vesicles for biomedical application particularly in the realm of cellular targeting [25,26,27].

**Figure 1:**
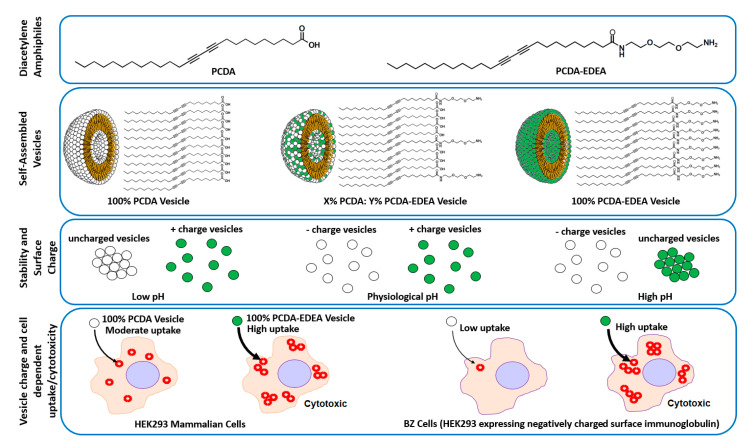
Overview of the use of diacetylene amphiphiles possessing either a carboxyl or amine terminal group for two component self-assembled vesicle formation. Surface charge of vesicles were found to be controllable as a function of pH and composition, and cell uptake and cytotoxicity were also determined to be cell and vesicle dependent.

In looking to a generalizable approach for controlling the surface charge of polydiacetylene vesicles in a pH responsive manner (Figure 1), we show here by modulating the composition of PCDA and PCDA–EDEA (ethylenedioxy-bis-ethylamine) amphiphiles that we may display acidic and basic groups, respectively. Thus, we can control the surface density and charge groups displayed by the vesicle surface as regulated by environmental pH. In doing so, we first identified regions of colloidal stability both visually and in relation to the zeta potential as a function of pH. Looking at various vesicle compositions, we examined cytotoxicity and interaction with cultured mammalian cells as a function of surface charge for the given vesicle compositions. Here, we examined the cellular association of the vesicles with amphiphile mixtures that provided a strongly cationic surface ultimately resulting in cell death. In addition, we observed that association of anionic vesicles with cells was inhibited by the display of negatively charged surface proteins on certain cell types, but the difference in cell surface proteins did not alter the cytotoxicity caused by cationic polydiacetylene vesicles. In the future, we may look to the possibility of using such charge selectivity for regulating cell uptake of vesicles only at low pH by using the appropriate composition of polydiacteylene amphiphiles, which may find applications in targeting the notably acidic tumor microenvironment [28].

## 2. Materials and Methods

### 2.1. PCDA–EDEA Synthesis

PCDA (10,12 pentacosadiynoic acid) monomer (purchased from Sigma Aldrich Inc., St. Louis, MO, USA) was dissolved in dichloromethane (DCM) and filter purified to remove polymerized components. Synthesis of the amine terminated amphiphile began from this pure monomer, which was coupled to 2,2′-(ethylenedioxy)bis(ethylamine) (EDEA) by using an EDC/NHS coupling strategy. In brief, the PCDA was reacted with 1.5 equivalents of 1-(3-dimethylaminopropyl)-3-ethyl carbodiimide (EDC) in DCM followed by 1.3 equivalents of N-hydroxysuccinimide (NHS) add dropwise and allowed to react for 2 h and 24 h, respectively. The DCM was removed by rotary evaporation and then extracted in a separatory funnel using a mixture of hexane, ethyl acetate, and water. The hexane/ethyl acetate phase was collected and again underwent rotary evaporation to obtain the PCDA-NHS product, which was dried, resuspended in DCM, and added dropwise to a flask containing EDEA (Scheme S1). After 24 h of reaction at room temperature, the mixture was rotary evaporated to remove DCM and then re-dissolved in a mixture of DCM containing 10% methanol and 1% ammonium hydroxide. After extraction against water, the solvent was collected and a final step of rotary evaporation was performed to collect the PCDA–EDEA product (Figure S1).

### 2.2. Fabrication of Polydiacetylene Vesicles

Vesicle mixtures comprised of distinct molar ratios of PCDA and PCDA–EDEA were prepared by dissolving 50 µmol of individual monomers to equivalent concentrations in DMSO and volumetrically controlling the ratio to produce either mixed or pure compositions in a final amount of 800 µL. For vesicle formation, this was added to 7 mL of heated 10 mM HEPES buffer and probe sonicated for 24 min followed by filtration and storage overnight at 4 °C to allow for self-assembly. The respective vesicles comprised of mixtures of 0%, 4%, 25%, 42%, 50%, 75%, or 100% PCDA–EDEA amphiphiles were polymerized using 254-nm UV light and stored at 4 °C until further analysis.

### 2.3. Zeta Potential Measurements

Zeta potential measurements were carried out using a NanoBrook 90 Plus PALS (Brookhaven, Holtsville, NY, USA). A square polystyrene cell (cuvette) was used for mixing the sample as well as for zeta potential measurements. The electrode assembly used was a BI-ZEL electrode and a constant temperature set at 25 °C. The run period was set at 3 cycles per measurement with a total of 6 measurements per sample. Particle concentration and diameter were determined simultaneously using dynamic light scattering. Twenty microliters of sample were exposed to UV irradiation at 254 nm for 45 min and this sample was mixed homogeneously with 1.5 mL of buffers varying in pH from 5 to 9 immediately prior to the reading.

### 2.4. Cell culture and Examination of Vesicle Association and Cytotoxicity

HEK293 cells were obtained from Dr. Kytai Nguyen and BZ cells were derived from this HEK293 cell line. These cell lines were cultured using standard cell culture techniques. Specifically, the cells were nourished with DMEM, 1% *v*/*v* penicillin/streptomycin, and 10% *v*/*v* newborn calf serum and maintained in an incubator at 37 °C in 5% CO_2_ with passaging every 2–3 days when reaching 80% confluence. Fluorescence and phase contrast microscopy was performed using 10×, 20×, and 40× objectives on an inverted Nikon Ti-2, with a Texas Red filter set. Nikon Elements software were used for acquiring the images. Fluorescence microscopy images were acquired for HEK and BZ cells after overnight culture in 24 well tissue culture plates with 500 µL of complete media (DMEM) and 1 µL of added vesicles with compositions as described above. The media was replaced with 500 µL of sterile phosphate buffered saline immediately prior to imaging to eliminate background fluorescence from the phenol red component of the DMEM media. Quantitative assessment of the red fluorescent polydiacetylene associated with the HEK and BZ cell lines was conducted by FACS (Flow Assisted Cells Sorting) analysis after overnight culture of the HEK and BZ cell lines with either PCDA or PCDA–EDEA vesicles. In brief, 500 µL of cells were cultured with 1 µL of vesicles as described above in 24-well plates overnight and on the following day washed with PBS immediately prior to resuspension in 500 µL of 0.1% BSA in PBS and filtered through a cell strainer prior to FACS analysis with a BD FACS Melody system. FACS data were analyzed using FlowJo software. Cytotoxicity studies were conducted by MTT assay as follows. In brief, 50 mg of MTT (methylthiazolyldiphenyl-tetrazolium bromide) purchased from Research Products International Corp were dissolved in 10 mL of PBS to serve as the MTT solution. HEK and BZ cells were grown overnight with controlled amounts of added PCDA or PCDA–EDEA vesicles in 96-well tissue culture plates in triplicate for each condition. After overnight culture, the media was replace with 100 µL of a mixture of 1 part DMEM containing no serum or antibiotic and 1 part MTT solution, and the 96-well plate was returned to the 37 °C incubator for 3 h followed by addition of 150 µL of MTT solvent (4 mMHCl and 0.1%NP40 in isopropanol). The plate was then shaken for 15 min at 200 rpm and the absorbance at 590 nm was recorded using an Epoch2 (BioTek) UV/Vis spectrophotometer plate reader. After subtraction of background signal from a well containing only the MTT solution, DMEM, and MTT solvent with no cells, the percent cell viability was calculated as: 100% × A590_Sample_/A590_Control_, where A590_Control_ is from the same cell type with no added vesicles.

## 3. Results and Discussion

**Figure 2:**
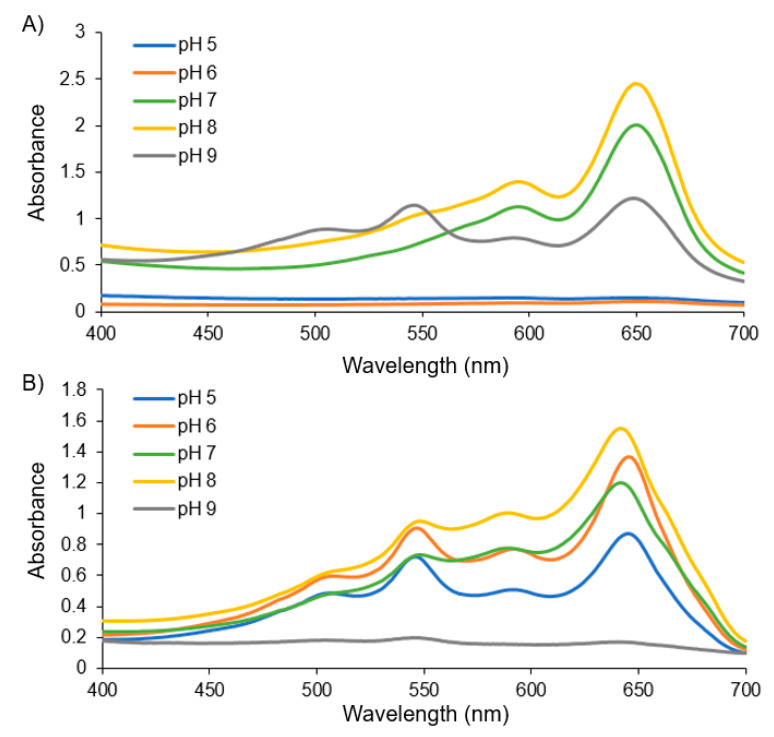
Spectrophotometer readings of (**A**) PCDA and (**B**) PCDA–EDEA vesicles after incubating in different pH environments.

Here, we describe the link that we identified between the relative proportions of PCDA vs. PCDA–EDEA content in a set of two-component vesicles with respect to vesicle surface charge and stability as a function of pH as well as their propensity for cell uptake and cytotoxicity. To begin, we synthesized the primary amine modified diacetylene monomer (PCDA–EDEA) to serve as the second amphiphile in conjunction with PCDA for our two-component vesicle system. In addition, two mixed component vesicles, pure PCDA vesicles, and pure PCDA–EDEA vesicles were also produced. Formation of polydiacetylene vesicles comprised of PCDA–EDEA, PCDA, or mixtures thereof was carried out by sonication of the component monomeric diaceytlene amphiphiles and subsequent self-assembly by overnight incubation at 4 °C followed by UV polymerization to cross-link the diacetylene amphiphiles. Examination of the vesicles by dynamic light scattering measurements revealed a diameter of approximately 175 nm, which is in the characteristic size range for polydiacetylene vesicles [29]. To begin exploring the stability of the vesicles possessing both PCDA and PCDA–EDEA components, we first generated vesicles with different molar ratios of PCDA and PCDA–EDEA amphiphile and examined the resulting vesicles after UV polymerization both visually for the appearance of aggregation and spectroscopically. Light microscopy of the vesicles suspensions at different pH values was conducted in a 96-well plate (Figure S3) showing a trend in the colloidal stability (or to say inversely, the aggregation behavior) in regards to the PCDA vs. PCDA–EDEA content relative to the environmental buffer pH. The specific link that we identified between composition and pH is that vesicles comprised purely of PCDA were unstable at low pH suffering aggregation but remained stable at even high pH. In contrast, vesicles produced with a mixture of PCDA–EDEA and PCDA became increasingly stable at low pH as a function of increasing PCDA–EDEA content but were interestingly found to instead aggregate at high pH. In looking closely at polydiacetylene vesicle fabrication when using a mixture of PCDA and PCDA–EDEA amphiphiles, we identified that different formulations exhibited distinct levels of stability which was tightly regulated by the pH. This lack of stability from the assembled vesicles at a 42% PCDA–EDEA:58% PCDA composition, which formed aggregates across the entire pH range examined. was a critical observation to direct our subsequent fabrication approach as such vesicles could be made stably when using an initial low pH buffer during fabrication. To summarize this link between colloidal stability and composition, vesicles which were predominantly comprised of PCDA–EDEA were found to be stable at lower pH ranges while aggregating above physiological pH. Inversely, vesicles comprised predominantly of PCDA were found to be stable at physiological pH and above, while aggregating at a lower pH. The aggregation and colorimetric response to different pH environments was also examined by measurement of the visible absorption spectra of the vesicles after incubation in pH environments from pH 5 to 9. From the spectra, we can see the typical maxima at ~650 nm for the vesicle suspensions when in the blue phase; however, aggregation resulted in a colorless background solution, as seen by attenuation in the absorption across all wavelengths. For example, in Figure 2, we see an attenuated spectra of PCDA vesicles at pH 5 and 6, while in contrast the PCDA–EDEA vesicles were attenuated at pH 9.

**Figure 3:**
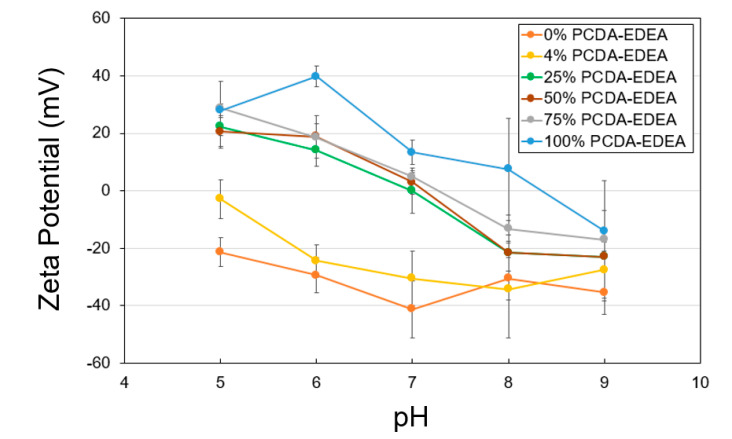
Zeta potential of polydiacetylene vesicles of different compositions showing the surface charge as a function of pH.

To shed light on the link between aggregation behavior in relation to the surface charge of the vesicles as dictated by the vesicle composition, we conducted zeta potential measurements of the distinct vesicle compositions as a function of pH and compared this to the visual and spectroscopic observations of stability described above. Pure PCDA vesicles appeared to have a negative surface charge which became more neutral at low pH and more negative at high pH, which is consistent with our observation of loss of colloidal stability (i.e., aggregation) at low pH. Conversely, the pure PCDA–EDEA vesicles exhibited a positive surface charge at low pH, which decrease to become neutral when at low pH. Again, this was consistent with our visual observations of pure PCDA–EDEA vesicles aggregation. Thus, we examined additional vesicles comprised of mixtures of PCDA and PCDA–EDEA and measured their surface charge while observing their corresponding colloidal stability. As shown in Figure 3, the PCDA:PCDA–EDEA vesicles exhibited a typical decrease in zeta potential with increasing pH; however, the rate at which this decrease occurred across pH was greatly amplified when the vesicles incorporated PCDA–EDEA. Increasing the proportion of PCDA–EDEA resulted in a shift of the isoelectric point of the vesicles to a higher pH. This correlates with visual observation of aggregation within the pH ranges at which the magnitude of the zeta potential was found to be minimal (less than ~15 mV) and thus of insufficient surface charge to stop vesicle aggregation. In contrast, we see stability of the suspension at pH values where the magnitude of the surface charge appears sufficient for repulsion between vesicles. The presence of a positive surface charge for vesicles comprised of PCDA–EDEA was identified for the lower pH range, which was to be expected as the population of primary amines presented by the PCDA–EDEA units on the vesicle become increasingly protonated as pH is lowered and thus resulting in a more cationic surface charge, while at higher pH becomes deprotonated and neutral. Conversely, the carboxyl group presented by the PCDA component is neutral when protonated to the neutral carboxylic acid, and it will become negatively charged when deprotonated as happens increasingly above the pKa. The pKa of the carboxylic acid and primary amine groups as presented on free individual molecules of PCDA and PCDA–EDEA are predicted to be 4.78 and 9.04, respectively; however, significantly higher effective pKa values of the carboxy side chains of polydiacetylene vesicles (up to 9.9) have been reported due to the confined side-chains causing close proximity of the carboxy groups at the vesicle surface [5]. During these experiments, we found that we could control the effective surface charge by modulating the relative content of PCDA–EDEA and PCDA comprising the vesicles, and as such we achieved vesicle compositions which would have the properties of a negative surface charge at pH 8 in order to maintain stability but which may switch to a positive charge upon decreasing the pH. An important consideration when making vesicles comprised of both PCDA and PCDA–EDEA arose when certain ratios of these amphiphile mixtures appeared to aggregate immediately after self-assembly, as we had seen for the case of 42% PCDA–EDEA due to the low magnitude of the surface charges at neutral pH. Thus, the original pH 7 buffer used in vesicle fabrication was not optimal for all compositions due to the apparent aggregation, and moreover the inability to re-suspend vesicles once aggregated. This problem was circumvented by conducting the vesicle fabrication at a reduced pH 4 when making vesicles with a PCDA–EDEA composition of 15–75%. This approach allowed us to ensure the stability of the as-prepared vesicles no matter the composition chosen for modulating the surface display of carboxyl or amine groups present on the surface, since this low pH facilitates sufficient positive surface charge character to provide repulsion between neighboring vesicles as to prevent aggregation.

**Figure 4:**
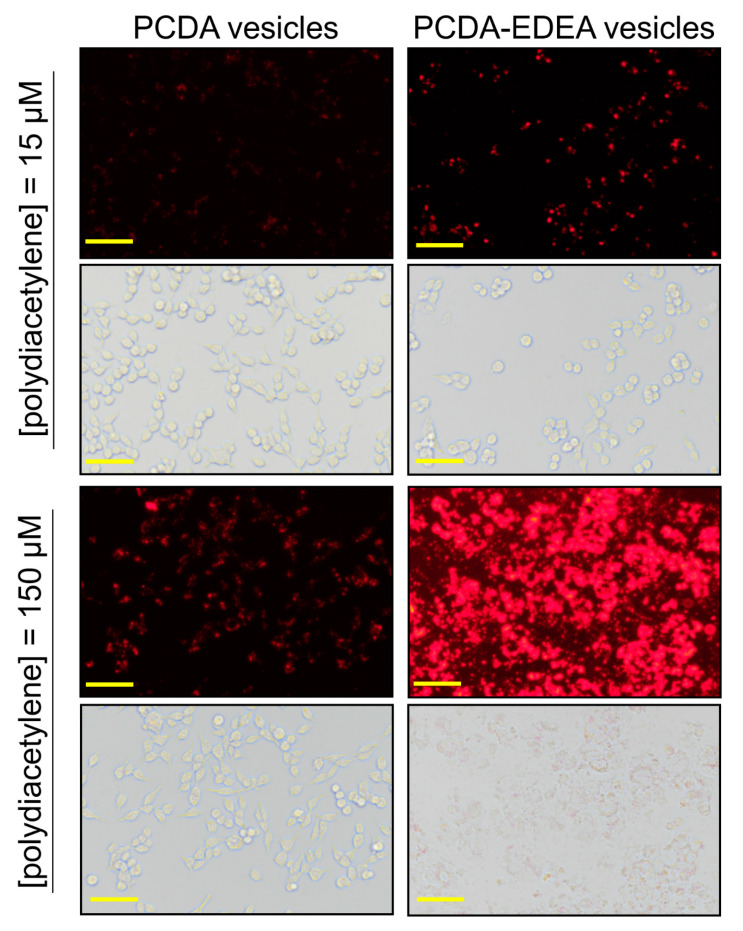
Fluorescence and phase contrast images of HEK293 cells cultured with PCDA or PCDA–EDEA vesicles at different concentrations revealing increased association and cell death for exposure to PCDA–EDEA notably when provided higher amounts of vesicles (scale bar: 50 µm).

The zeta potential results indicate that increasing the PCDA–EDEA content of the vesicles would increase the net surface charge distribution by virtue of the primary terminal amine of PCDA–EDEA, which is in contrast to the carboxyl group of the PCDA monomer that would decrease the effective net surface charge. Based on the well-known ability of cationic particles to be taken up into mammalian cells (as is exploited by transfection reagents), we next explored if our vesicles would be taken up by mammalian cells, and so we examined cell cultures containing our vesicles comprised of different proportions of PCDA vs. PCDA–EDEA. By virtue of the natural red fluorescence of the polydiacetylene vesicles, we were able to use fluorescent microscopy to directly observe the vesicles when associated or taken up by the human embryonic kidney cells (HEK293). Observing the fluorescence arising from the vesicles, we could see a very clear increase in the degree of association of the fluorescent vesicles with the HEK293 cells and a correspondingly higher cytotoxicity when providing PCDA–EDEA vesicles at increased concentrations as compared to PCDA vesicles (Figure 4). To clarify, when incubating the HEK cells with 15 µM of PCDA vesicles or 15 µM of PCDA–EDEA vesicles, we observe an increased amount of vesicles as indicated by the brighter fluorescence at the location of the cells for the case of PCDA–EDEA as compared to PCDA, which suggests greater cell uptake by the cationic PCDA–EDEA vesicles than the anionic PCDA vesicles. This was to be expected given that it is well known that positively charged nanoparticles are internalized by cells to a greater extent than negatively charged nanoparticles [30]. In addition, increasing the amount to 150 µM of vesicles we could see significant cell death for HEK cells incubated with the PCDA–EDEA vesicles but not for PCDA vesicles, where cationic nanoparticles are believed to be more cytotoxic due to their ability to cause markedly higher levels of plasma-membrane disruption than anionic nanoparticles [30].

**Figure 5:**
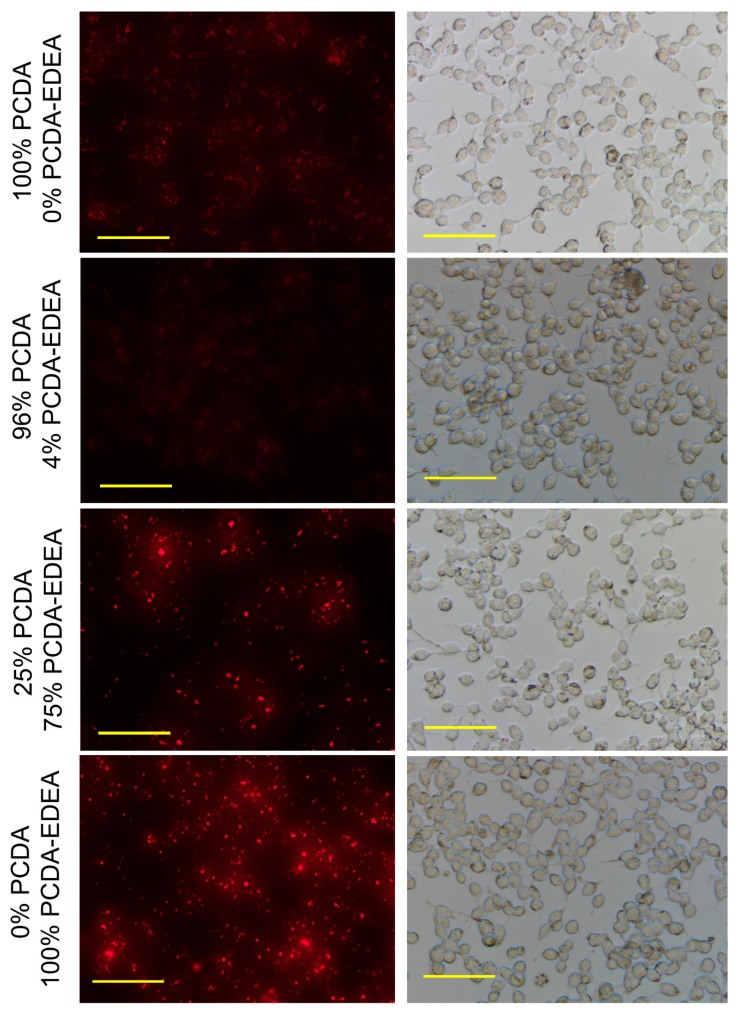
Microscopy images revealing fluorescence from endocytosed vesicles for HEK293 cells cultured with PCDA–EDEA:PCDA vesicles of different compositions: 0% PCDA–EDEA; 4% PCDA–EDEA; 75% PCDA–EDEA; and 100% PCDA–EDEA (scale bar: 50 µm).

When examining vesicles containing increasing PCDA–EDEA composition, we observed an associated increased fluorescence associated with the HEK293 cells (Figure 5). Here, we see the prospect of tuning of vesicle association by controlling the PCDA–EDEA composition; we identified that 75% PCDA–EDEA vesicle exhibited a cell association behavior at physiological pH but to a lesser extent than the 100% PCDA–EDEA vesicles. Anionic vesicles comprised of 0% or 4% PCDA–EDEA had very low association. Of course, the association of cationic vesicles with mammalian cells was to be expected as poly-cationic polymeric nanoparticles such as polyethyleneimine are used extensively for transfection of genes into cells. The interesting feature here is thus the control of vesicle surface charge by modulating their composition of amino-functionalized vs. carboxyl-functionalized diacetylene amphiphile monomers. In addition, the behavior of positive charged particles in interacting with mammalian cells in vitro is also known such that they are able to deposit in tissue and be taken up into cells, while neutral or anionic particles are transient and have only minimal deposition [31,32,33]. This work has however opened the prospect of optimization of conditions to promote cell association of our vesicles under a reduced pH environment. Our rationale for future work was to examine the prospect of local deposition of vesicles at a low pH tumor site by utilizing surface charge mediated cellular interaction of our vesicles. This reasoning is based on the known occurrence of extracellular acidosis in certain tumor microenvironments as a result of altered metabolism where breast cancers have measured to be as low as pH 6.44 [28,34,35]. We thus examined if our vesicles may be able to switch from anionic at physiological pH to cationic at reduced pH, which we found to be the case. Careful examination of fluorescence microscopy images of the HEK293 cells after incubation with anionic PCDA vesicles or cationic PCDA–EDEA vesicles revealed a distinct difference in the fluorescence distribution across the cells depending on the type of vesicle. Specifically, we could consistently observe (Figure S4) a moderate fluorescence that was dispersed throughout the entire space occupied by the HEK293 cells after incubation with PCDA vesicles, whereas the fluorescence appeared heterogeneously clustered only at distinct regions of the HEK293 cells when incubated with PCDA–EDEA vesicles. Interactions with the cell surface caused those PCDA as well as PCDA–EDEA vesicles to switch to the red fluorescent state appearing as sources of red fluorescence.

**Figure 6:**
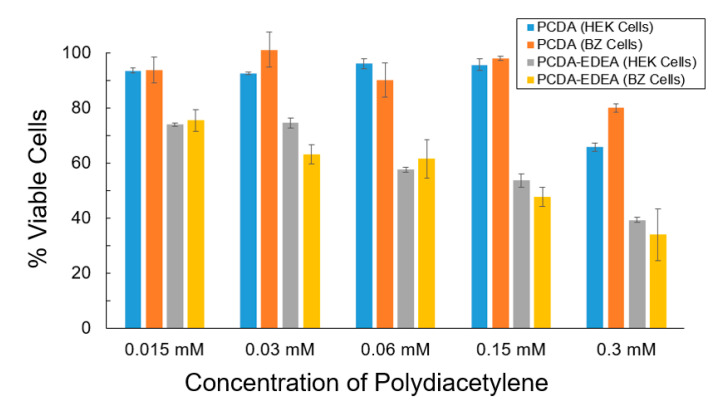
Examination of the percent cell viability (100% × A590_Sample_/A590_Control_) of the polydiacetylene vesicles comprised of PCDA or PCDA–EDEA compositions for both HEK293 and BZ cells.

To more clearly examine the cell viability for PCDA and PCDA–EDEA vesicles, we conducted MTT assays using two cell types, specifically HEK293 as well as a derivative we refer to as BZ which expresses a negatively charged surface antibody (Figure 6). We see that the PCDA vesicles had little impact on the cell viability of the HEK293 and BZ cells, while the PCDA–EDEA was considerably cytotoxic even at low concentrations. There was no apparent visual difference in cell viability when comparing cells with and without vesicles comprised of 100% PCDA; however, visual inspection of cultures provided vesicles comprised of PCDA–EDEA appeared to cause noticeable cell death relative to control cultures. This corresponds well with microscopic images showing positively charged PCDA–EDEA vesicles accumulating as large distinct clusters within the HEK cells. Correspondingly, MTT assay of cells culture with PCDA–EDEA showed these vesicle formulations to be more cytotoxic than PCDA vesicles. This was to be expected since at physiological pH the PCDA–EDEA vesicles display many protonated amino groups that cause a high cationic charge density. In looking at the wealth of literature regarding positively charged nanoparticles, polyethyleneimine and others are often used as transfection reagents for delivering genetic cargo to mammalian cells, where they undergo endocytosis and can be strongly cytotoxic at appreciable concentrations [36,37]. The mechanism of cytotoxicity from such positively charged nanoparticles has been proposed to be an inactivation of certain intracellular enzymes and increase in reactive oxygen species [38,39,40]. Because we could effectively tune the isoelectric point, we can control the vesicle surface charge for different compositions as a function of pH and thus provide a means for controlling the cytotoxicity.

To put these observation in context, existing studies of ratios of binary mixtures of phospholipids containing one saturated phospholipid and one photo-polymerizable lipid have afforded critical information including the observation that photo-polymerizable liposomes display enhanced membrane rigidity as the photo-polymerizable lipid content is increased [41]. In that same study, the authors also identified that domains dominated by either the photo-polymerizable or saturated lipid co-exist within the liposomes and will interact distinctly depending on the chain length of the saturated phospholipid component [42]. Specifically, they suggest that using a saturated phospholipid component with shorter chain length can provide better distribution of the photo-polymerizable component but in turn limit the degree of cross-linking, where this could conceivably be used to tune the leakiness for drug delivery applications. Looking into reports on mechanisms by which polymerizable lipid particles may be taken up into cells by endocytosis, we found studies of polydiacetylene nanoparticles in which the head group was controlled to explore positive, neutral, or negatively charged particle uptake into cells [16]. In their report, the speed of internalization was fastest for positive polydiacetylene particles and slowest for negatively charged particles, which they proposed to be a result of charge interactions with negatively charged phospholipids on the cell surface. In comparing our results, we also find that the positively charged PCDA–EDEA vesicles have the highest association with both the HEK and BZ cell lines, while the negatively charged PCDA vesicles have lower association with HEK and almost no association with the BZ cell line that expressed the negatively charged surface immunoglobulin. The biophysicochemical interactions that modulate the endocytosis of such particles are numerous and the complexity is outside the scope of this article; however, to highlight relevant literature, it has been proposed that the composition of the protein corona that adsorbs onto nanoparticles having different charge can have a critical role in determining how that nanoparticle interacts with the cell surface ultimately affecting endocytic uptake and if so by which mechanism [43]. Studies of endocytosis of polydiacetylene nanoparticles revealed these to be primarily via energy-dependent processes with polydiacetylene found to be taken up predominantly by caveolae-mediated endocytosis [16] wherein FACS was used to determine the extent of internalized polydiacetylene. In looking at this in relation to our results, we may consider again that the BZ cell line exhibited decreased association of PCDA vesicles as compared to the HEK cell line as a result of the BZ cells overexpression of negatively charged membrane bound antibodies. The fact that caveolins, which are needed to form the caveolae invagination for endocytosis, are also membrane proteins, and the literature shows that transmembrane proteins are largely excluded from caveolae-mediated endosomal compartments [44,45]. Hence, our observation may be the result of the presence of antibodies displayed in the BZ cell membrane directly limiting the extent of caveolae-mediated endocytosis by competing for space with caveolin. Interestingly, no difference in uptake was observed between BZ and HEK for the positively charged PCDA–EDEA vesicles but only between BZ and HEK cells incubated with negatively charged PCDA vesicles, suggesting either a different uptake mechanism or that indeed it was the negative charge as presented by the surface antibodies that repelled the negatively charged PCDA vesicles from associating with the BZ cells. While there continue to be discrepancies in the literature as to the effect of size on uptake by caveolae, some reports suggest this mechanism for particles in the 200–500-nm range [42,46]. We did not examine the effect of size in this study, but it is worth noting that the PCDA vesicles possessed a diameter of 175 nm (Figure S2).

**Figure 7:**
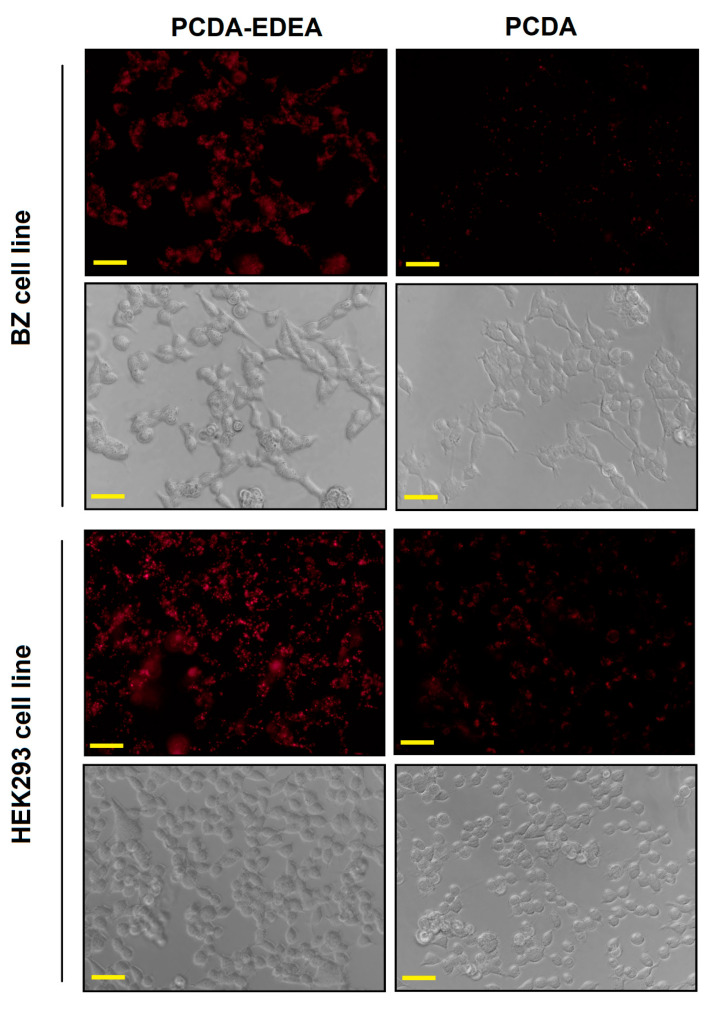
Fluorescence microscopy and phase contrast microscopy images of HEK293 and BZ cell lines exposed to PCDA or PCDA–EDEA vesicles. BZ cells which are engineered to express and display a negatively charged surface immunoglobulin appear to have less interaction with PCDA vesicles in contrast to the background HEK293 cell line (scale bar: 20 μm).

**Figure 8:**
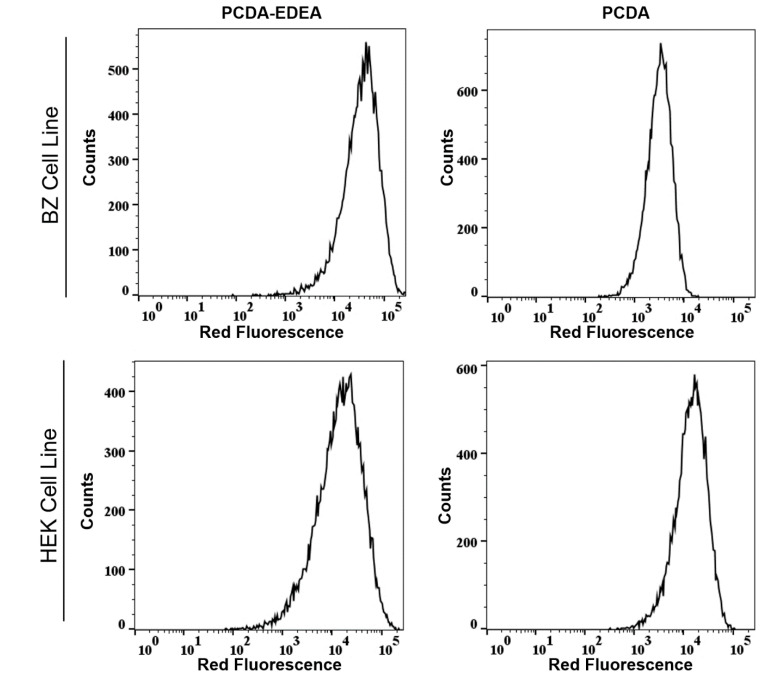
Flow cytometry histograms reveal that the BZ cell line had a much lower amount of associated fluorescent PCDA vesicles as compared to the HEK293 cell lines, as indicated by the order of magnitude lower fluorescence intensity.

Validation of the different interactions between the vesicles and the two cell types was also confirmed by flow assisted cell sorting (FACS), where we found an enhancement in the mean fluorescence intensity of PCDA exposed to HEK293 cells as compared to BZ cells (Figure 7), while the PCDA–EDEA response was consistent between the two cell types with slightly higher uptake of PCDA–EDEA by the BZ cells compared to the HEK293 cells. The importance of the BZ cell line in this case is that it is a HEK293 background engineered to overexpress a membrane bound antibody (surface immunoglobulin) wherein the antibody possesses a negative charge under physiological conditions. An interesting observation was made when comparing the association of vesicles taken up by HEK293 cells versus the BZ cell line, which again could be determined by the red fluorescence intensity by FACS as well as by the degree of fluorescent at the location of the cells when examined by fluorescence microscopy. Specifically, the PCDA vesicles were not taken up by the BZ cell line to the same extent as the HEK293 cells, as confirmed by fluorescent microscopy and flow cytometry (Figure 7 and Figure 8). We may suspect this to be the result of the negatively charged immunoglobulin expressed on the BZ cell surface providing a means for electrostatic repulsion of the negatively charged PCDA vesicles. A direct comparison of the overlaid FACS histograms can be seen in the Supplementary Materials (Figure S5), clearly revealing a greater amount of the negatively charged PCDA vesicles being associated with the HEK cell line as compared to the BZ cell line. Conversely, HEK293 and BZ cells had similar levels of PCDA–EDEA vesicle uptake with slight enhancement for the BZ cell line that may be related to electrostatic attraction between the negatively charged surface immunoglobulin and the cationic PCDA–EDEA vesicles.

## 4. Conclusions

We identified an interesting behavior of the vesicles in terms of their surface charge as a function of pH and their corresponding proportion of PCDA to PCDA–EDEA within these two-component vesicles. It was determined that the vesicle surface charge could be controlled through tuning of the relative PCDA to PCDA–EDEA content for a given pH, and as such we were able to identify a set of vesicle compositions that could maintain colloidal stability within a desired pH range. Specifically, by incorporating PCDA–EDEA content to an increasing proportion of the vesicle composition, we found the vesicles to maintain colloidal stability within more acidic conditions which was not the case for the purely PCDA vesicles. From zeta potential measurements, this can be attributed directly to the increase in surface charge at lower pH environments as resulting from protonation of the primary amine of PCDA–EDEA. Furthermore, when exploring the different vesicles incubated in mammalian culture with the HEK cell line, it was found that the pure PCDA–EDEA vesicles were cytotoxic and exhibited a fluorescence within the cell that was distinctly heterogeneous when compared to incubation with purely PCDA vesicles which were relatively non-cytotoxic and exhibited a more homogeneous fluorescence across the cells. Interestingly, expression of a negatively charged antibody on the surface of a related cell line, referred to as BZ, provided a very different behavior in which there was greatly reduced uptake of pure PCDA vesicles, presumably resulting from charge repulsion of these negatively charged PCDA vesicles, as compared to pure PCDA incubated with the HEK cell lines. Correspondingly, the positively charged pure PCDA–EDEA vesicles were taken up significantly by the BZ cell line as well as the HEK cell line. In summary, the aggregation vs. colloidal stability behavior of the mixed component vesicles could be controlled by modulating the pH with respect to the PCDA vs. PCDA–EDEA content of the vesicles, and quantitative assessment of vesicle zeta potentials revealed this to be a factor of changes in the effective surface charge which beyond a certain threshold prevented aggregation. Finally, the prospect that polydiacetylene vesicles could be controlled in terms of their cell association and cytotoxicity by simply controlling the PCDA vs. PCDA–EDEA content in this two-component system was a significant step forward. We hope the results revealed in this study will be of practical interest for biomedical applications of targeting vesicles to the acidic tumor microenvironment in future works, and further efforts by our group toward utilizing such mixed component vesicle to modulate the association of vesicles with cells in a pH dependent manner is also underway.

## Supplementary Materials

The following are available online at https://www.mdpi.com/2079-6374/10/10/132/s1, Figure S1: ^1^H NMR spectrum of PCDA-EDEA (300 MHz, CDCl_3_) including solvent peaks at 7.3 ppm (CHCl_3_) and 1.7 ppm (H_2_O); Figure S2. Log normal dynamic light scattering graph of PCDA vesicles; Figure S3. Microscopy images of vesicles comprised of different ratios of PCDA-EDEA: PCDA and examined for aggregation behavior in different pH environments within a 96 well plate (scale bar: 100μm); Figure S4. High magnification fluorescence microscopy and phase contrast images of HEK293 cells after overnight incubation with 100% PCDA vesicles (top) and 100% PCDA-EDEA vesicles (bottom) revealing a distinct difference in distribution of the red fluorescent vesicles throughout the cell (scale bar: 10 μm); Figure S5. FACS histograms normalized to mode for BZ cells incubated with PCDA vesicles (orange) and HEK cells incubated with PCDA vesicles (green) revealing the preferential association of the negatively charged PCDA vesicles with the HEK cell line as compared to the BZ cell line as revealed by the enhanced red fluorescence of the PCDA vesicles; Scheme S1. Synthesis of PCDA-EDEA monomer.
