# Supplementary material for: Tuning the Surface Charge of Self-Assembled Polydiacetylene Vesicles to Control Aggregation and Cell Binding
Source: Biosensors (Basel). 2020 Sep 24;10(10):132. doi: 10.3390/bios10100132 (PMC7598607; doi:10.3390/bios10100132)
Supplement: Supplementary file 1 [file biosensors-10-00132-s001.pdf]

biosensors

MDPI

Supplementary Information

Synthesis of PCDA-NHS:

PCDA (10,12-pentacosadiynoic acid) monomer (500 mg, 1.33 mmol) is dissolved in 15mL of methylene chloride. EDC (1-Ethyl-3-(3-dimethylaminopropyl) carbodiimide) (374 mg, 1.95 mmol) is dissolved in 15 mL of methylene chloride then added dropwise with stirring to the PCDA in a round bottom flask and covered with foil for stirring at room temperature for 2 hours. NHS (N-hydroxysuccinimide) (195 mg, 1.69 mmol) was dissolved in 20mL of methylene chloride then added dropwise to the PCDA-EDC mixture at room temperature with stirring and allowed to stir covered for 24 hours. The mixture was then rotary evaporated to remove the methylene chloride and the product was dissolved in 250 mL of a 3:1 (hexane:ethyl acetate) solvent mixture and moved to a separatory funnel for extraction against 250mL of water keeping the solvent phase and repeated several times. The solvent was then transferred to a round bottom flask and rotary evaporated to remove the solvent. The product was then dissolved in methylene chloride and transferred to an amber flask followed by evaporation of the methylene chloride to provide 495mg (1.05mmol) of pure PCDA-NHS (yield 79%).

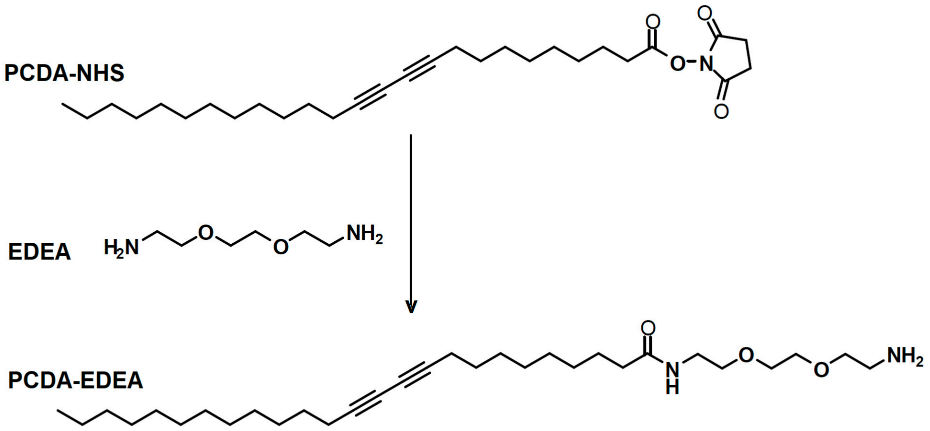

Scheme S1. Synthesis of PCDA-EDEA monomer.

Synthesis of PCDA-EDEA: PCDA-NHS (205mg, 0.44mmol) was dissolved in methylene chloride (20mL) and added dropwise to a stirred round bottom flask containing EDEA (2,2-(ethylenedioxy) bis (ethylamine)) (300mg, 2mmol) dissolved in 30mL of methylene chloride at room temperature and was reacted overnight. The mixture was rotary evaporated and the product was dissolved in 250 mL of methylene chloride containing 10% methanol and 1% ammonium hydroxide and moved to a separatory funnel for extraction against 250mL of water keeping the solvent phase and repeated several times before transfer to a rotary evaporator for removal of solvent to provide 153mg (0.3mmol) of pure PCDA-EDEA (yield 68%).

Characterization: ^{1}H spectra were measured with a Jeo ECX-300 MHz NMR. For particle size measurements, 10uL of PCDA vesicles suspensions in 10mM HEPES buffer were added to 3mL of DI water and mixed thoroughly before placing in a cuvette and into the chamber of the NanoBrook 90Plus PALS instrument. For DLS measurement parameters, the temperature was set at 25°C and the run period was set at 6 cycles per measurement.

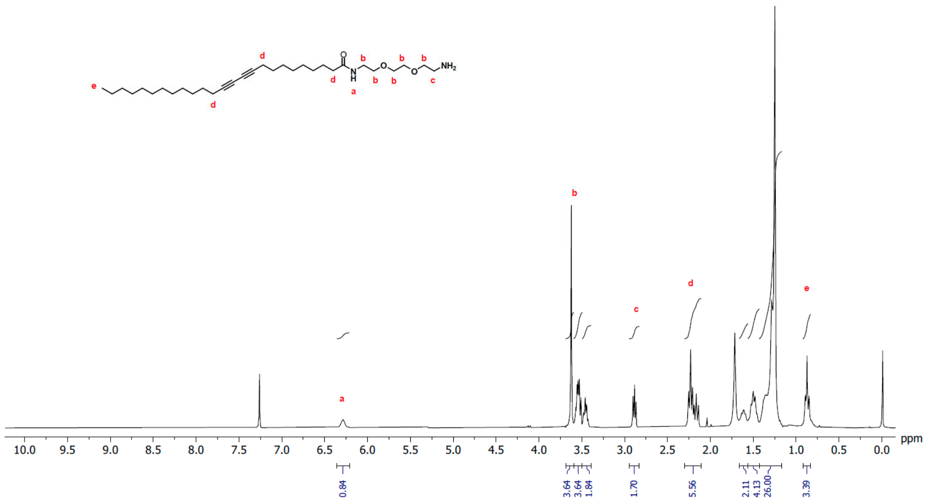

Figure S1. ^{1}H NMR spectrum of PCDA-EDEA (300 MHz, $CDCl_3$) including solvent peaks at 7.3 ppm ($CHCl_3$) and 1.7 ppm (H_2O).

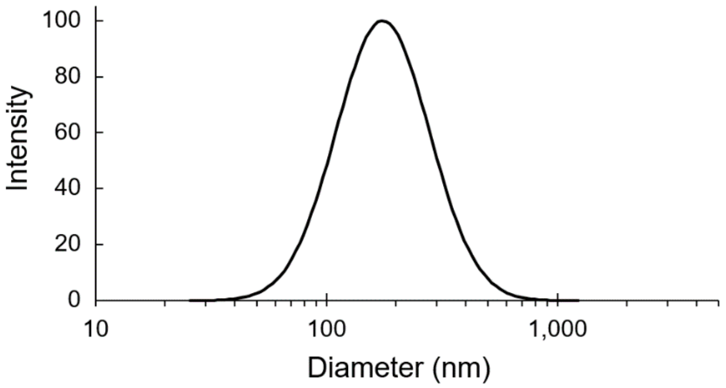

Figure S2. Log normal dynamic light scattering graph of PCDA vesicles.

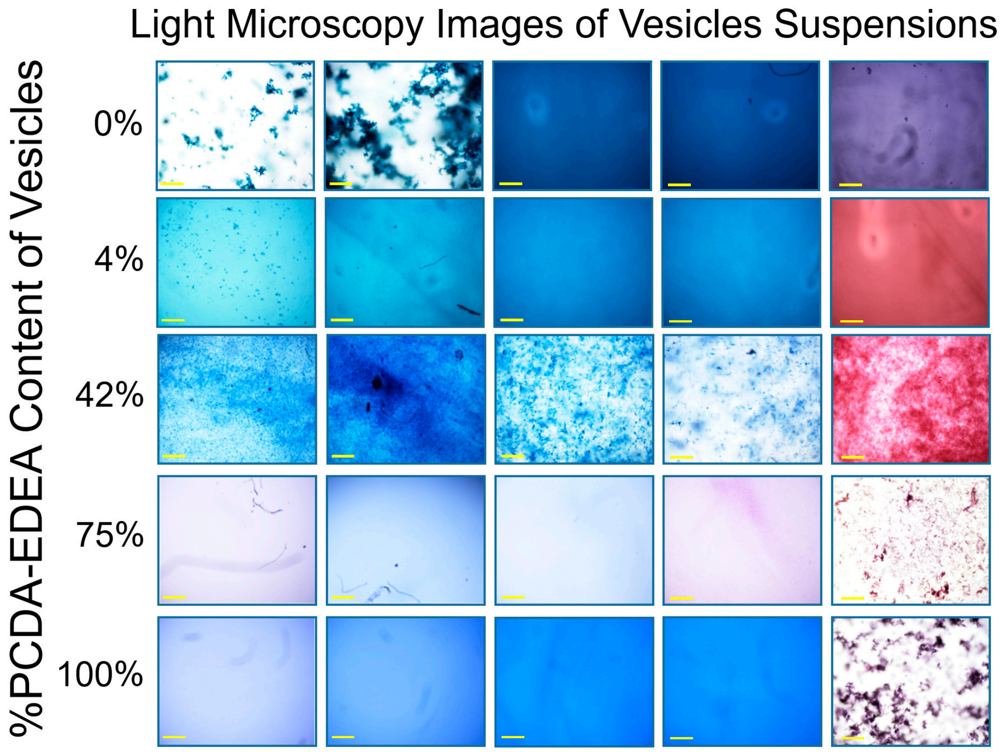

Figure S3. Microscopy images of vesicles comprised of different ratios of PCDA-EDEA: PCDA and examined for aggregation behavior in different pH environments within a 96 well plate (scale bar: 100um).

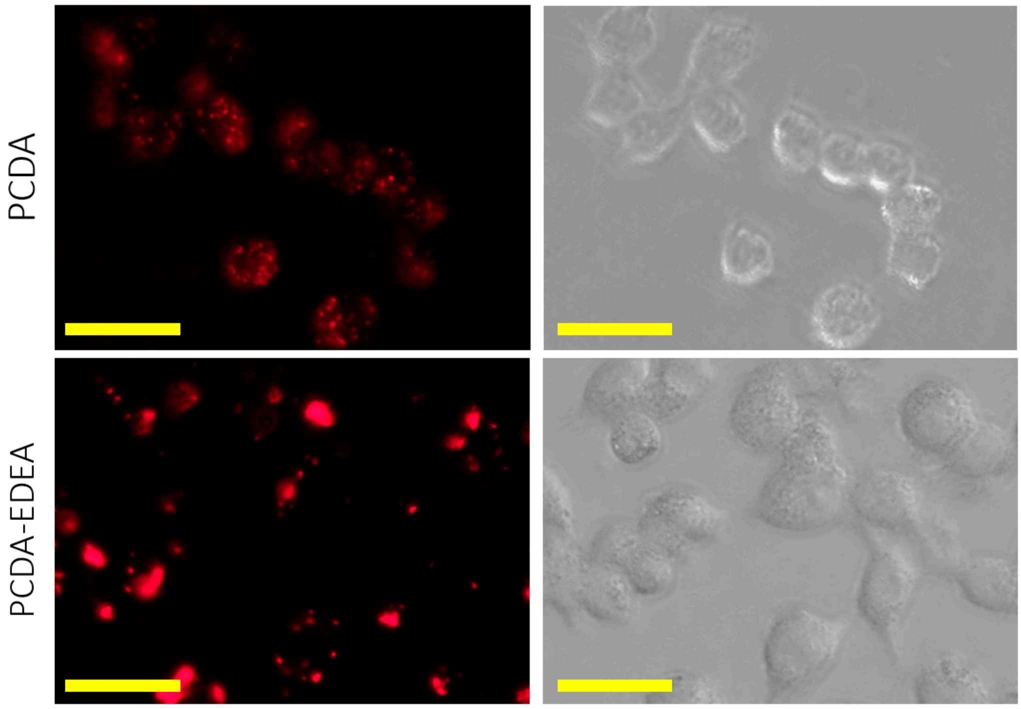

Figure S4. High magnification fluorescence microscopy and phase contrast images of HEK293 cells after overnight incubation with 100% PCDA vesicles (top) and 100% PCDA-EDEA vesicles (bottom) revealing a distinct difference in distribution of the red fluorescent vesicles throughout the cell (scale bar: 10um).

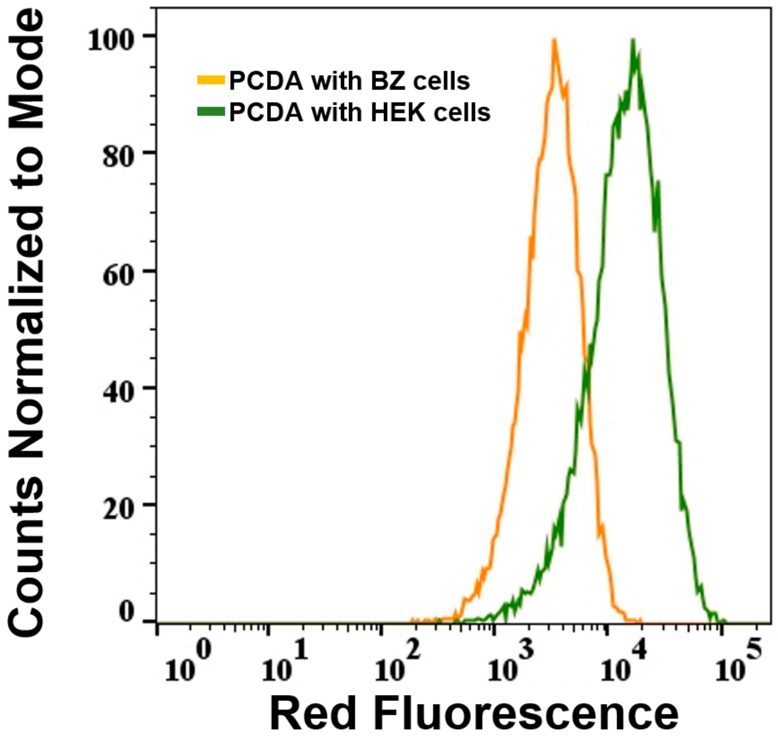

Figure S5. FACS histograms normalized to mode for BZ cells incubated with PCDA vesicles (orange) and HEK cells incubated with PCDA vesicles (green) revealing the preferential association of the negatively charged PCDA vesicles with the HEK cell line as compared to the BZ cell line as revealed by the enhanced red fluorescence of the PCDA vesicles.
